# Improvement of Testicular Steroidogenesis Using Flavonoids and Isoflavonoids for Prevention of Late-Onset Male Hypogonadism

**DOI:** 10.3390/antiox9030237

**Published:** 2020-03-13

**Authors:** Luc J. Martin, Mohamed Touaibia

**Affiliations:** 1 Biology Department, Université de Moncton, Moncton, NB E1A 3E9, Canada; 2 Chemistry and Biochemistry Department, Université de Moncton, Moncton, NB E1A 3E9, Canada; Mohamed.Touaibia@umoncton.ca

**Keywords:** testis, testosterone, androgen, Leydig cells, polyphenols, flavonoids

## Abstract

Androgen production, being important for male fertility, is mainly accomplished by the Leydig cells from the interstitial compartment of the testis. Testosterone plays a critical role in testis development, normal masculinization, and the maintenance of spermatogenesis. Within seminiferous tubules, appropriate Sertoli cell function is highly dependent on testicular androgen levels and is essential to initiate and maintain spermatogenesis. During aging, testosterone production by the testicular Leydig cells declines from the 30s in humans at a rate of 1% per year. This review outlines the recent findings regarding the use of flavonoids and isoflavonoids to improve testosterone production, contributing to normal spermatogenesis and preventing age-related degenerative diseases associated with testosterone deficiency. With the cumulation of information on the actions of different flavonoids and isoflavonoids on steroidogenesis in Leydig cells, we can now draw conclusions regarding the structure-activity relationship on androgen production. Indeed, flavonoids having a 5,7-dihydroxychromen-4-one backbone tend to increase the expression of the steroidogenic acute regulatory protein (StAR), being critical for the entry of cholesterol into the mitochondria, leading to increased testosterone production from testis Leydig cells. Therefore, flavonoids and isoflavonoids such as chrysin, apigenin, luteolin, quercetin, and daidzein may be effective in delaying the initiation of late-onset hypogonadism associated with aging in males.

## 1. Introduction

Testicular Leydig cells are the main site of androgen synthesis in mammals. Androgens are responsible for the development and maintenance of male sex organs and secondary sex characteristics. Specifically, embryonic testosterone is involved in the development of Wolffian ducts, leading to the formation of the epididymis, vas deferens, and seminal vesicles. In addition, testosterone is indirectly involved in the development of the genital tubercle to form the penis, scrotum, and prostate by its conversion to 5 α-dihydrotestosterone (DHT), the most potent androgen of the body. At puberty, testosterone plays a major role in masculinization of the brain, development of male sexual behavior, initiation and support of spermatogenesis, bone growth, maturation of the external genitalia, hair growth, and regulation of gonadotropins (luteinizing hormone (LH) and follicle-stimulating hormone (FSH)) secretion. Within the seminiferous tubules, Sertoli cells are stimulated by androgen through the expression of the androgen-binding protein (ABP, SHBG). In turn, activated Sertoli cells support germ cell division and normal spermatogenesis to maintain fertility. With aging, testosterone production declines, beginning in the 30s in men, at a rate of 1% per year [1]. Different conditions, such as exposure to endocrine disruptors, obesity, diabetes and cancer treatments, exacerbate this decrease in serum testosterone levels, leading to the development of late-onset hypogonadism [2]. Age-related functional alterations include decreased response of testicular Leydig cells to the pituitary LH [3]. In addition, middle-aged men have an increased prevalence of obesity and insulin resistance associated with low serum total testosterone and SHBG levels [4]. These men are mainly responsible for the increase in testosterone-replacement therapies over the last decade [5]. Men with androgen deficiency may present with decreased libido, erectile dysfunction, decreased body hair, increased fatigue, decreased muscle mass and strength, increased body fat, low bone mineral density, irritability, inability to concentrate, and poor quality of life [6,7]. Therefore, therapies aiming to increase serum levels of testosterone may contribute to relieving part of these symptoms and may involve nutritional supplementation with flavonoids. In this review, a structure-activity relationship for plant polyphenols with flavonoid backbone on testosterone production will be investigated to draw conclusions from cumulated literature thus far.

## 2. Steroidogenesis

Within the testis, androgen production, of which testosterone is the most abundant, occurs almost exclusively in Leydig cells. Androstenedione and dehydroepiandrosterone (DHEA) are also produced by the testis. However, their levels of androgenicity are lower than those of testosterone. Adult Leydig cells do express the aromatase enzyme (CYP19A1) which is responsible for the conversion of androgen into estrogen [8]. However, this rate of conversion of testosterone into estradiol is minimal compared to the secretion levels of androgen, and estrogen may instead play a buffering action on the steroidogenesis by Leydig cells [9].

**Figure 1:**
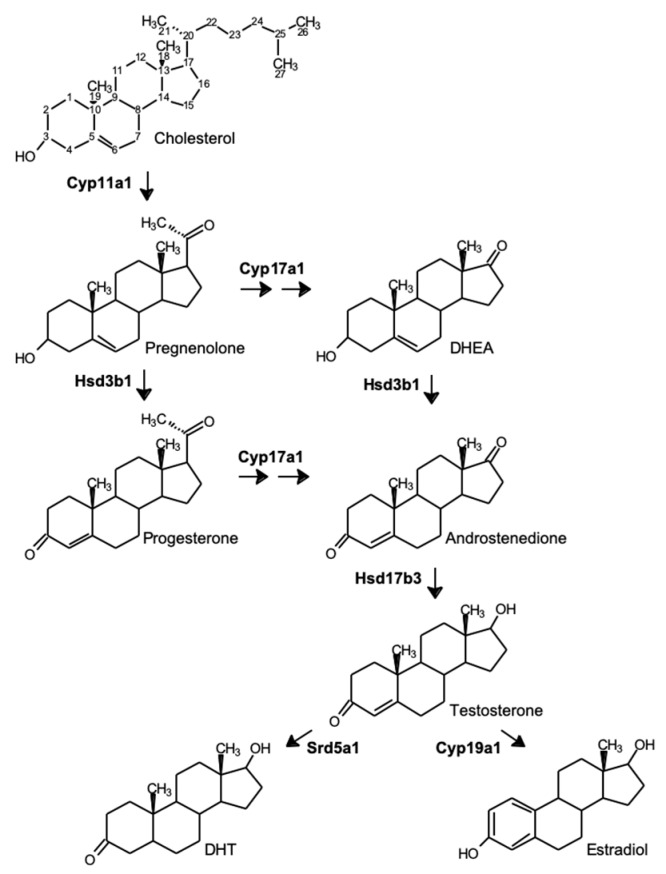
Common pathways for steroid synthesis within Leydig cells. Abbreviations for enzymes: Cyp11a1, P450 side-chain cleavage; Hsd3b1, 3β-hydroxysteroid dehydrogenase; Cyp17a1, P450 17α-hydroxylase/20-lyase; Hsd17b3, 17β-hydroxysteroid dehydrogenase; Srd5a1, 5α-reductase; Cyp19a1, P450 aromatase. Abbreviations for steroids: DHEA, dehydroepiandrosterone; DHT, dihydrotestosterone.

Testosterone synthesis from cholesterol involves the sequential action of several steroidogenic enzymes such as the cholesterol side-chain cleavage enzyme (CYP11A1), cytochrome P450 17α-hydroxylase/20-lyase (CYP17A1), 3β-hydroxysteroid dehydrogenase (HSD3B1 in rodents and HSD3B2 in humans), and 17β-hydroxysteroid dehydrogenase type 3 (HSD17B3), catalyzing a cascade of hydroxylation, cleavage, dehydrogenation, and isomerization reactions. Testosterone synthesis from cholesterol and its conversion to other estrogenic and androgenic active metabolites is presented in Figure 1.

In Leydig cells, as in other steroidogenic tissues, cholesterol is the primary substrate for steroid synthesis. Cholesterol may be synthesized from acetyl-coenzyme A or derived from plasma reserves through the receptor-mediated endocytosis of low-density lipoprotein (LDL) particles. In each case, cholesterol is stored in esterified form in cytoplasmic lipid droplets. The contribution of the two sources of cholesterol for steroidogenesis varies according to cell type, species, physiological status, and availability. Under normal physiological conditions, Leydig cells depend mainly on the endogenous production of cholesterol for testosterone synthesis. However, increased cholesterol demand can be fulfilled by increased extracellular cholesterol uptake from LDL and low-density lipoprotein (HDL) using the LDL receptor and Scavenger receptor class B, type I (SR-BI) surface receptors [10].

Translocation of cytoplasmic cholesterol to the inner mitochondrial membrane is initiated by the recruitment and activation of members of a carrier protein complex, including the steroidogenic acute regulatory protein (StAR) and the translocator protein (TSPO), also known as the peripheral benzodiazepine receptor, at the outer mitochondrial membrane [11,12]. Once inside the mitochondria, cholesterol is converted to pregnenolone by the CYP11A1 enzyme through side-chain (C21-C27) cleavage (Figure 1). This reaction also involves the electron carrier ferredoxin and NADPH: ferredoxin reductase as the electron donor [13]. To break the side chain of cholesterol, CYP11A1 hydroxylates the C22 and C20 of cholesterol to produce 22R-hydroxycholesterol and 20α,22R-dihydroxycholesterol, respectively. Afterward, CYP11A1 cuts the side chain of 20,22-dihydroxycholesterol to release pregnenolone (of 21 carbons) and a 6-carbon aldehyde [13]. The high affinity of CYP11A1 for 22R-hydroxycholesterol and 20α,22R-dihydroxycholesterol, along with very high rates of conversion, prevents the accumulation of these intermediates within the mitochondria. Once synthesized, pregnenolone dissociates from the active site of CYP11A1 to exit the mitochondria by diffusion and reaches the smooth endoplasmic reticulum (SER) where androgen synthesis is completed. Three enzymatic reactions involving HSD3B, CYP17A1, and HSD17B3 contribute to complete androgen synthesis. The HSD3B enzyme has dehydrogenase and isomerase activities as it is involved in the oxidation of the hydroxyl group on C3 to a ketone moiety and in the formation of a double bond at C5 of pregnenolone to form progesterone [14]. The CYP17A1 enzyme is then able to catalyze the hydroxylation of progesterone on C17 to generate the 17α-hydroxyprogesterone, followed by the cleavage of the C17-C20 bond to form androstenedione. Thereafter, the enzyme HSD17B3 catalyzes the conversion of androstenedione into testosterone using NADPH as a cofactor. Once produced, testosterone will be used to promote the initiation and maintenance of spermatogenesis within the testis as well as the development of male secondary sex characteristics.

### Regulation of Steroidogenesis

The expression of genes encoding the steroidogenic enzymes within Leydig cells is primarily regulated by the LH, which activates the cyclic adenosine monophosphate (cAMP)/Protein kinase A (PKA) signalling pathway. Binding of the LH to its G-protein-coupled membrane receptor (LHCGR) leads to the activation of adenylate cyclase, conversion of ATP to cAMP, and subsequent activation of PKA. Substrates for PKA involve the StAR, responsible for the transport of cholesterol inside mitochondria, and transcription factors critical for steroidogenic gene expressions. In addition to the cAMP/PKA pathway, other signalling pathways such as the mitogen-activated protein kinase (MAPK), Janus kinase/signal transducer and activator of transcription proteins (JAK/STAT), calcium/calmodulin-dependent protein kinase (CAMK), and protein kinase C (PKC) have been shown to influence steroidogenesis from testicular Leydig cells. Indeed, activation of the epidermal growth factor receptor (EGFR) contributes to the regulation of steroidogenesis by the modulation of MAPK signalling and its downstream extracellular-signal-regulated kinases 1 and 2 (ERK1/2) [15,16]. The JAK/STAT pathway has been shown to be critical for the modulation of steroidogenesis from Leydig cells by adipose-derived hormones such as leptin and resistin [17,18]. Increased intracellular Ca^2+^ levels result in increased concentrations of Ca^2+^ complexed to calmodulin, leading to the activation of the Ca^2+^/calmodulin kinase kinase (CAMKK) and its downstream target, Ca^2+^/calmodulin kinase I (CAMKI). CAMKI is expressed in adult Leydig cells and has been shown to be activated downstream of cAMP and to be essential for the *StAR* gene expression [19]. Others have shown that the co-activations of PKC and PKA play an important role in upregulation of StAR and steroidogenesis in Leydig cells [20,21]. In addition to the modulation of intracellular-signal transduction pathways, paracrine factors may also be shared through cell junctions and participate in the regulation of steroidogenesis within Leydig cells. Moreover, nutrients such as polyphenolic compounds found in plants may promote testosterone production through different regulatory mechanisms. Among these compounds, certain flavonoids have been shown to influence testosterone production and such action may depend on specific structural features.

## 3. Flavonoids

**Figure 2:**
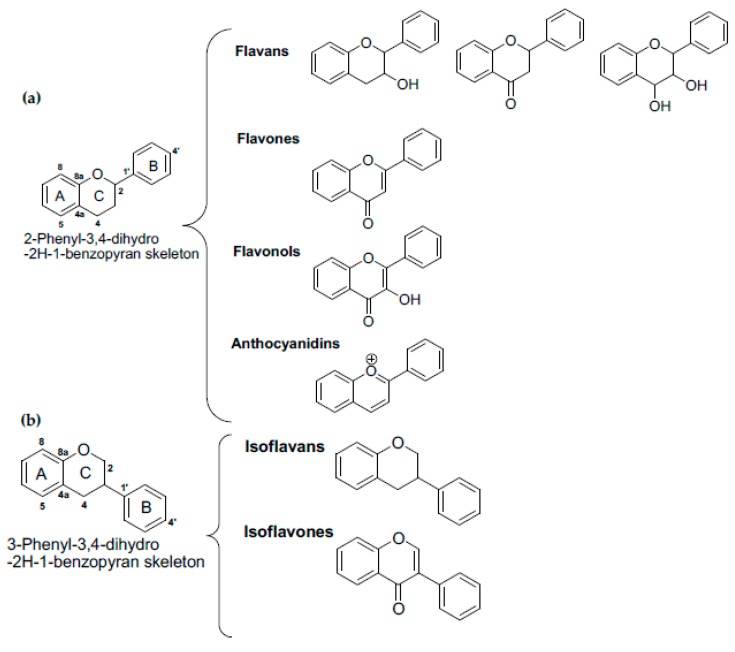
Classification of major groups of flavonoids (**a**) and isoflavonoids (**b**).

Flavonoids are characterized by the C6-C3-C6 group in which two benzene rings are connected by a three-carbon segment. The structure of the different flavonoid types varies at the level of the oxygenated heterocycle formed by the chain of three carbons between the two benzene rings and hydroxyl (-OH) moieties of the A ring (see Figure 2 for a description of the structures of flavonoids found in plants). The structure of the C3 region varies by its level of oxidation which is highest in flavonols. Catechins and dihydrochalcones are the most reduced flavonoids. On the other hand, the structure of the flavonoids corresponding to the minimum oxidation state—that is, to a complete opening and hydration of the heterocycle between the two flavonoid rings (A and B)—is not known in nature.

Based on the 2-Phenyl-3,4-dihydro-2H-1-benzopyran skeleton, there are four major groups of flavonoids (Figure 2a): flavans, flavones, flavonols, and anthocyanidins [22]. Isoflavonoids are divided into two main families (Figure 2b): isoflavans and isoflavones, both based on the 3-Phenyl-3,4-dihydro-2H-1-benzopyran skeleton [22]. The flavones are characterized by a planar structure attributed to the double bond in the central aromatic ring. Examples of flavones include apigenin and luteolin, found in parsley, thyme, and celery. The isoflavones are similar in structure, except that the B ring is associated with the carbon 3 rather than carbon 2 of the C ring. Examples of isoflavones are genistein and daidzein, found in soybeans. Flavonols is an important group of flavonoids characterized by the presence of a hydroxyl group at the position-3 of the C ring. Quercetin, myricetin and kaempferol, found in onions, apples, broccoli, cherries, tea, and berries, are members of this group. Flavanones such as naringenin and hesperedin, characterized by the absence of a double bond within the C ring, are mainly found in citrus fruits and plums. The flavonoids belonging to the catechin group, characterized by the absence of a double bond within the C ring and the presence of a hydroxyl group at the position-3 of the C ring, are mainly found in tea, apples, and red wine. Anthocyanidins such as cyanidin and delphinidin, characterized by the presence of two double bonds and a positive charge on the C-ring, are found in strawberries and other berries, such as blueberries and blackberries, in wine, and tea.

### 3.1. Flavonoids and Steroidogenesis

Since their discovery, flavonoids have been associated with numerous health benefits including cancer prevention, reduced risk of cardiovascular and neurodegenerative diseases, and delayed-aging-associated symptoms (reviewed in [23]). Having a chemical structure similar to cholesterol and other steroids, flavonoids may influence the production of androgens in Leydig cells. Therefore, more than 500 publications have reported effects of different flavonoids on testosterone production since the early 1960s. However, it is only recently that the molecular mechanisms of flavonoids affecting steroid synthesis have been partially elucidated.

#### 3.1.1. Flavones

**Figure 3:**
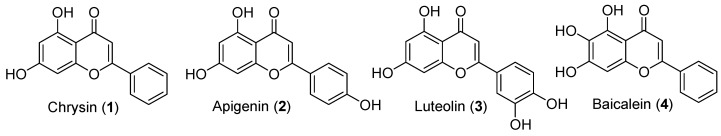
Structures of chrysin (**1**), apigenin (**2**), luteolin (**3**), and baicalein (**4**).

Differently from falvans, flavones are characterized by the presence of the C2-C3 double bond which is conjugated with the C4 carbonyl (Figure 2). In one of our studies, we found that 10 µM of chrysin (**1**), apigenin (**2**), luteolin (**3**), and baicalein (**4**) (Figure 3) stimulated cAMP-dependent *StAR*, *Cyp11a1*, and *Fdx1* (Ferredoxin 1) expression in MA-10 Leydig cells [24]. The presence of the two hydroxyls in positions 5 and 7 (cycle A) and one or two hydroxyls in positions 3′ and 4′ (cycle C) are crucial for the biological effect of these molecules. Compared to chrysin (**1**), baicalein (**4**) has an extra hydroxyl in position 6 of the A cycle.

Only luteolin (**3**) was able to increase the cAMP-dependent accumulation of progesterone from MA-10 Leydig cells, possibly through its positive regulation of *StAR* expression. Luteolin (**3**) also activated *StAR* expression and resulted in increased progesterone and testosterone syntheses in LC540 tumor Leydig cells [25]. Apigenin (**2**) and chrysin (**1**) have been shown to increase cAMP-dependent androgen production from testicular Leydig cells by increasing the *StAR* gene expression [26,27].

As for chrysin (**1**), apigenin (**2**) was able to decrease the levels of DAX1 (dosage-sensitive sex reversal, adrenal hypoplasia critical region, on the X chromosome, gene 1), an important repressor of *StAR* transcription [27]. In addition, apigenin (**2**) also inhibits COX2 (cyclooxygenase-2) expression, which may contribute to increased *StAR* expression. Increased *Cox2* expression is associated with decreased expression of *StAR* and reduced testosterone production in Leydig cells from aging males [28]. However, others have shown that apigenin (**2**) inhibited the production of 5α-androstane-3α, 17β-diol (DIOL), the main androgen of rat immature Leydig cells [29]. The activity of rat Hsd3b, Cyp17a1, and Hsd17b3 was inhibited by apigenin (**2**) with IC_50_ values in the 10 µM range [29]. In addition, human HSD3B2 and HSD17B3 were more than five times more sensitive to the inhibitory action of apigenin (**2**). Using H295R human adrenal cells, treatments with 10 µM apigenin for 24h resulted in a decrease of androstenedione and testosterone production [30,31]. In this cell model, apigenin (**2**) decreased the expressions of *HSD3B2* and *CYP17A1* [31]. Overall, apigenin (**2**) seems to increase steroidogenesis in Leydig cells mainly by increasing PKA-dependent StAR protein expression. However, such effect may not translate into increased testosterone production as we have also reported that apigenin (**1**) inhibits *Cyp11a1* expression in LC540 tumor Leydig cells [25].

Luteolin (**3**), a flavone, has been of growing interest in our laboratory for its potential in improving androgen production. Indeed, we have shown that luteolin (**3**) can increase Leydig cell steroidogenesis by upregulating StAR expression [24,25]. We also reported that luteolin (**3**) was able to activate *Fdx1* (Ferredoxin 1) transcription in MA-10 Leydig cells [24]. Fdx1 is important to supporting the Cyp11a1 steroidogenic enzyme activity by electron transfer. Similar to apigenin (**2**) and chrysin (**1**), luteolin (**3**) is also able to inhibit Cox2 expression, thus promoting *StAR* transcription [32]. Others have also reported that luteolin (**3**) is able to increase cAMP-dependent steroidogenesis by improving mitochondrial import of cholesterol by increasing StAR protein levels in MA-10 Leydig cells [33]. Furthermore, luteolin (**3**) increased *StAR* transcription by inhibiting the expression of *Dax-1* as reported for other flavonoids [33].

Chrysin (**1**), 5,7-dihydroxyflavone, is present in high levels in honey, propolis, chamomile, mushrooms, fruit bark, and many plant extracts. Chrysin (**1**) increased cAMP-dependent StAR protein levels and steroidogenesis in mouse MA-10 Leydig cells, possibly by decreasing the expression of Dax1 [26]. Interestingly, chrysin (**1**) seems to increase the sensitivity of Leydig cells to cAMP-dependent stimulation of *StAR* expression. Chrysin (**1**) is also able to inhibit the activity of the transcription factor NF-κB, leading to reduced *COX2* promoter activity [34]. Lower levels of Cox2 in Leydig cells contributes to increased *StAR* expression. In addition, others have shown that chrysin (**1**) is a potent inhibitor of the enzyme aromatase, which converts testosterone into estradiol [35]. This inhibitory effect of human aromatase activity has been assigned to different flavonoids in addition to chrysin (**1**) and apigenin (**2**) [36]. Therefore, chrysin (**1**) increases testosterone serum levels by more than 35% in adult male rats [37]. However, chrysin (**1**) supplementation did not change urine testosterone levels in humans after 21 days of treatment [38]. This may be attributed to the relatively low dose of chrysin (**1**) used in this research compared to studies with rodents. However, treatments of male mice for 10 days with up to 20 mg/kg of chrysin (**1**) had no effect on serum testosterone levels, while preventing the inhibition of testosterone production by exposure to the mycotoxin zearalenone [39]. Therefore, it has been suggested that chrysin (**1**) could be used to delay age-related decline in StAR expression and testosterone production from Leydig cells [26,39,40]. Importantly, chrysin (**1**) failed to induce a significant increase in steroid production when MA-10 Leydig cells were co-incubated with 22(R)hydroxycholesterol [26], suggesting this flavonoid only improves the entry of cholesterol into the mitochondria by regulating StAR protein levels and has no effects on steroidogenic enzyme activity.

Baicalein (**4**) is an important flavonoid found in several plants such as roots of *Scutellaria baicalensis* Georgi [41] and *Oroxylum indicum* [42]. As for other flavones, we have shown that low concentrations of baicalein (**4**) increased cAMP-dependent *StAR* promoter activation in MA-10 Leydig cells [24]. In addition, *Cyp11a1* and *Fdx1* promoter activity was also enhanced by co-treatments with cAMP as reported for apigenin (**2**), luteolin (**3**), and chrysin (**1**). However, others recently reported that baicalein (**4**) administration for four weeks reduced serum testosterone and FSH and LH levels following treatments of a rat model for polycystic ovary syndrome (PCOS) [43]. In their study, baicalein (**4**) instead decreased the expression of *StAR*, *Hsd3b*, *Cyp11a1,* and *Cyp19a1* in ovarian tissues. Such discrepancy in responses to baicalein (**4**) may be attributed to the pathological condition of PCOS and different responses to baicalein (**4**) according to the sex of animals. Overall, flavones seem to have a stimulatory effect on the *StAR* gene expression in Leydig cells. However, such action may not always translate into increased testosterone production.

#### 3.1.2. Isoflavones

**Figure 4:**
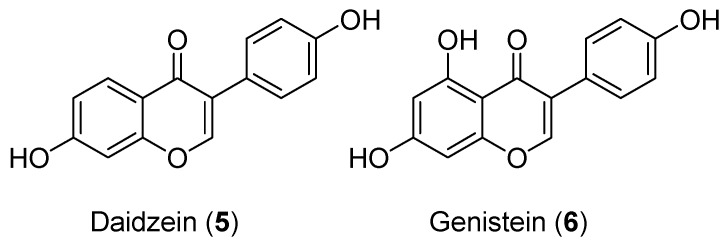
Structures of daidzein (**5**) and genistein (**6**).

In mice, estrogen receptor (ER)-β is detected in every cell type of the testis, whereas ER-α is mainly expressed by Leydig cells [44]. Expressing the enzyme aromatase, Sertoli cells synthesize estrogen from androgen [45], which has an inhibitory effect on steroidogenesis from Leydig cells [46]. However, since adult Leydig cells produce higher levels of estrogen [47], the physiological significance of estrogen-mediated regulatory interactions between Sertoli cells and Leydig cells remains to be better defined. Having phytoestrogenic effects, isoflavones such as daidzein (**5**) and genistein (**6**) (Figure 4) may disrupt the paracrine signalling by estrogen between Sertoli and Leydig cells. The 5-hydroxyl substitution (cycle A) is crucial since it is the only difference between these two molecules. Indeed, daidzein (**5**) impairs Leydig cell testosterone production by inhibiting the expression levels of StAR, Cyp11a1, and Hsd3b1 in neonatal mouse testes [48]. Genistein (**6**) inhibited progesterone secretion through the down-regulation of *StAR* expression in MA-10 Leydig cells [49]. Others have reported that genistein (**6**) exposure was also able to inhibit testosterone production from fetal mouse testes by decreasing StAR, Cyp11a1, Hsd3b1, and Cyp17a1 expression levels [50]. Genistein also inhibited HSD3B and HSD17B3 enzyme activities from rat and human testes [51]. therefore, high levels of isoflavones may have detrimental effects on testicular steroidogenesis during the early neonatal period.

Although numerous studies have suggested that a phytoestrogenic action of isoflavones contributed to decreased testosterone levels in men, it was concluded from a meta-analysis including 32 reports that neither soy foods nor isoflavone supplementation had a significant effect on bioavailable testosterone levels [52]. In addition, treatments of MA-10 Leydig cells with 0.1-100 µM genistein (**6**) for 48h had no effect on cell viability, basal progesterone synthesis, and the expression of steroidogenic genes such as *StAR*, *Tspo*, *Cyp11a1,* and *Hsd3b1*, while decreasing hCG-stimulated progesterone production when combined with the phtalate MEHP [53]. However, perinatal exposure of male rats to soy isoflavones induced proliferation of Leydig cells and increased the levels of StAR, CYP11A1, HSD3B, and CYP17A1, compensating for the decrease in HSD17B3 steroidogenic-enzyme activity [54]. Moreover, exposure to genistein (**6**) during perinatal development in male rodents resulted in reduced anogenital distance, lower testosterone serum levels, and decreased testicular mass compared to male rats under a normal diet [55,56,57]. In addition, low doses of 10 nM genistein (**6**) inhibits testosterone secretion by fetal Leydig cells through interaction with the estrogen receptor α and reduction of StAR and steroidogenic-enzyme gene expression during early fetal development (E12.5), a critical period for male programming [50]. Therefore, fetal exposure to phytoestrogen may disturb the development and function of the male reproductive system.

Genistein (**6**) and daidzein (**5**) have been reported to inhibit the activity of 5α-reductase in vitro [58] and to reduce the plasma levels of 5α-dihydrotestosterone (DHT) in male rats [59]. Dihydrotestosterone is the main prostatic androgen responsible for the development of prostate cancer [60]. Interestingly, lifetime exposure to flavonoids such as daidzein (**5**) and genistein (**6**) increased serum and testicular testosterone levels in rats [61].

#### 3.1.3. Flavonols

**Figure 5:**
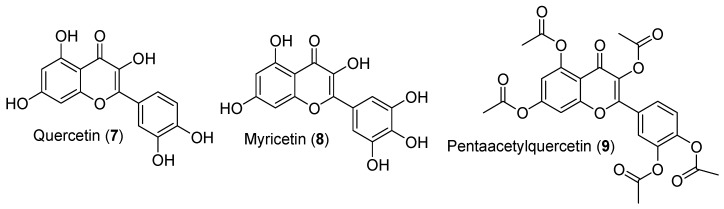
Structures of quercetin (**7**), myricetin (**8**), and pentaacetylquercetin (**9**).

Flavonols, especially quercetin (**7**), have been well documented for their positive effects on testicular function and steroidogenesis. Indeed, treatments of MA-10 Leydig cells with 10 µM of quercetin (**7**), myricetin (**8**), or pentaacetylquercetin (**9**) (Figure 5) resulted in increased cAMP-dependent expressions of *StAR*, *Cyp11a1,* and *Fdx1*, contributing to increased steroidogenesis and accumulation of progesterone [62]. However, there were no changes in serum testosterone levels following quercetin (**7**) supplementation of healthy men for eight weeks [63]. Such discrepancy in the effects of quercetin on testosterone production may be attributed to differences between species.

Quercetin (**7**), which does not have an extra hydroxyl in the position 5′ of the B-cycle as in myricetin (**8**), has been shown to improve steroidogenesis and testosterone levels in male mice exposed to bisphenol A, widely used in the production of plastics [64]. However, the molecular mechanism responsible for such improvement may be attributable to the antioxidative properties of quercetin (**7**). Supporting its positive effect on steroidogenesis, quercetin improves *Creb1* transcriptional activity, as well as *Cyp11a1* and *Fdx1* promoter activity [62]. Creb1 is an important activator of steroidogenic-gene expression, including *StAR*, in Leydig cells [65,66,67]. Others have also reported that quercetin (**7**) increased *StAR* mRNA levels, *StAR* promoter activity, and steroid hormone production from MA-10 Leydig cells [49]. Quercetin (**7**) may increase *StAR* gene expression in response to cAMP stimulation by reducing Dax1 protein levels in Leydig cells [33]. The expression of *StAR* and steroidogenesis are also increased by blocking Cox2 signalling in response to quercetin (**7**), as well as chrysin (**1**), apigenin (**2**), and luteolin (**3**) treatments of Leydig cells [33]. Quercetin (**7**) also contributes to the improvement of testosterone production in rats exposed to either cadmium chloride or the herbicide atrazine, where the enzyme activities of Hsd3b and Hsd17b3 are recovered [68,69]. Indeed, we have shown that quercetin (**7**) can activate the expression of *Hsd3b* in rat LC540 tumor Leydig cells [25]. An acetylated form of quercetin (**7**), pentaacetylquercetin (**9**), successfully increased cAMP-dependent accumulation of progesterone from MA-10 Leydig cells, possibly through the activation of *StAR* and *Cyp11a1* transcriptions [62]. Such chemical modification may improve the bioavailability of quercetin (**7**) in vivo.

**Figure 6:**
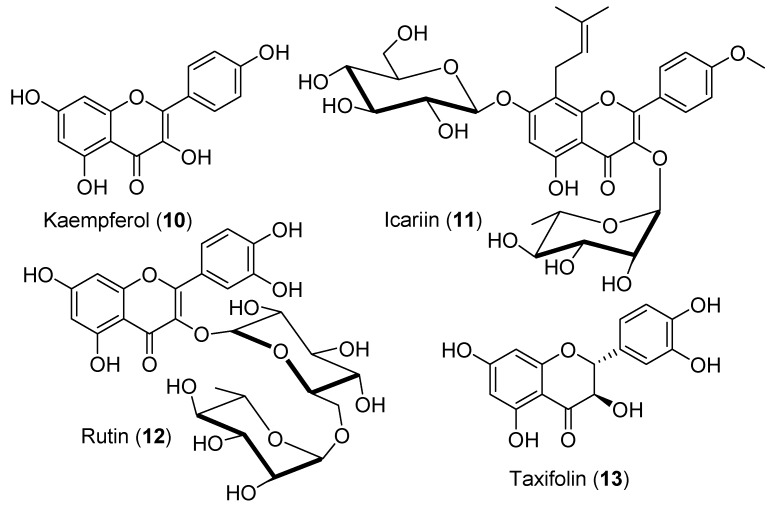
Structures of kaempferol (**10**), icariin (**11**), rutin (**12**), and taxifolin (**13**).

Icariin (**11**) is a prenylated flavonol glycoside (Figure 6) derived from kaempferol (**10**) and isolated from several species of plant belonging to the genus *Epimedium,* such as the horny goat weed. Icariin (**11**) has been shown to reverse the adverse effects of di(2-ethylhexyl) phtalate (DEHP) on primary Leydig cell proliferation and testosterone synthesis [70]. Specifically, icariin (**11**) allowed to recover from the inhibitory effects of DEHP on the expression of steroidogenic enzymes (Cyp11a1, Hsd3b1, and Hsd17b3) and the transcription factor Nr5a1 (Sf-1), known to regulate steroidogenic gene expression. In addition, icariin (**11**) also contributed to the increase of the expressions of peripheral-type benzodiazepine receptor (PBR) and steroidogenic acute regulatory protein (StAR), leading to increased testosterone production in adult-male rat testes [71]. However, others have reported that icariin (**11**) induces apoptosis in mouse Leydig tumor cells (mLTC1) [72], suggesting an anticancer potential.

Recently, rutin (**12**) (Figure 6), a glycosylated quercetin (**7**) found in citrus fruits, was shown to reverse the decreased levels of serum testosterone, LH, and FSH, as well as the impaired sperm quality, induced by carbon tetrachloride in male rats [73]. Carbon tetrachloride is an important environmental contaminant inducing male hypogonadism. Rutin (**12**) also partially reversed cadmium-induced decline in plasma testosterone levels in male rats by increasing Hsd3b and Hsd17b3 enzyme activity [74,75].

Taxifolin (**13**) (Figure 6), also known as dihydroquercetin, belongs to the subclass of flavanonols and is found in red onions. Taxifolin (**13**) has been shown to inhibit androgen production in immature rat Leydig cells by inhibiting the activities of Hsd3b and Cyp17a1 enzymes [76]. However, human HSD3B2 and CYP17A1 were less sensitive to taxifolin (**13**) compared to rat enzymes. Overall, flavonols such as quercetin (**7**) are promising in increasing steroidogenic enzymes activities and may prevent age-related decline in testosterone production in men.

#### 3.1.4. Flavanones

**Figure 7:**
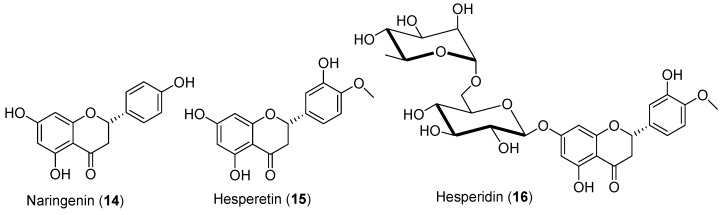
Structures of naringenin (**14**), hesperetin (**15**), and hesperidin (**16**).

Naringenin (**14**, Figure 7), a flavanone found in grapefruits, has been reported to inhibit the activity of Hsd17b3 and Hsd3b in male rats after daily subcutaneous injections of 15 mg/kg [77]. However, these changes in enzyme activity had no consequences on testosterone levels. Oppositely, oral administration of naringenin (**14**) to male rats for 10 weeks resulted in increased serum testosterone levels [78]. Therefore, the route of delivery of flavonoids may influence the result outcome in certain experimental designs. Naringenin (**14**) also prevented the decreases of serum testosterone and inhibin B in rats receiving chemotherapeutic drugs such as cisplatin and doxorubicin [79]. Hesperidin glycoside, found in citrus fruits such as lemon and oranges, has been reported to reverse vanadium-induced decline in testosterone serum levels in male rats [80]. Hesperidin (**16**), a glycosylated form of hesperetin, (Figure 7) was also reported to reverse the decline of testosterone production in diabetic rats [81]. Therefore, flavanones may have the potential to prevent testosterone decline in response to endocrine disruptors, and possibly to aging.

#### 3.1.5. Catechins

**Figure 8:**
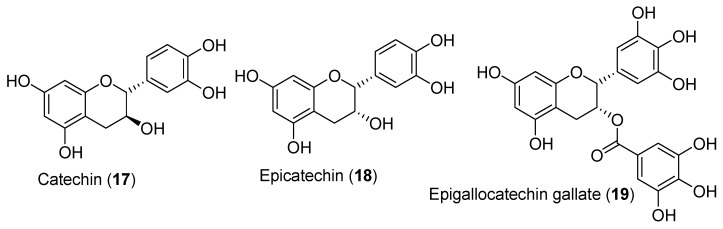
Structures of catechin (**17**), epicatechin (**18**), and epigallocatechin gallate (**19**).

In male rats, catechin (**17**), epicatechin (**18**), and epigallocatechin gallate (EGCG, **19**) (Figure 8) have been shown to increase plasma testosterone levels after only 8h of treatment [82]. Moreover, catechins were effective in increasing human chorionic gonadotropin (hCG)-stimulated testosterone production from purified rat Leydig cells [82]. Specifically, epicatechin (**18**), which is a catechin (**17**) epimer, increased the activity of Hsd17b3. However, there is evidence that green tea polyphenols have an inhibitory effect on testosterone production from rat Leydig cells by inhibiting the PKA/PKC pathways, Cyp11a1, and Hsd17b3 [83]. In addition, chronic green tea consumption was also associated with decreased plasma testosterone levels due to enhanced aromatase expression [84]. However, oral administration of catechin (**17**) resulted in increased plasma testosterone levels in male rats by inhibiting aromatase activity [85]. Moreover, injections with catechins or its derivatives also increased testosterone plasma concentrations [82]. Such discrepancy in the effects of catechins on testosterone levels needs further investigation to better define their regulatory mechanisms.

EGCG (**19**), isolated from green tea, has been reported to inhibit estradiol and progesterone production by swine granulosa cells [86]. In addition, EGCG (**19**) inhibits testosterone production from both basal and hCG-stimulated primary Leydig cells without affecting cell viability [83]. In this study, they found that EGCG (**19**) inhibited the PKA/PKC signalling pathways and decreased the enzyme activity of Cyp11a1 and Hsd17b3. Due to the important variability in the effects of catechins on androgen production, more research should be performed to better define the potential these molecules have to maintain testosterone levels according to aging.

#### 3.1.6. Anthocyanidins

**Figure 9:**
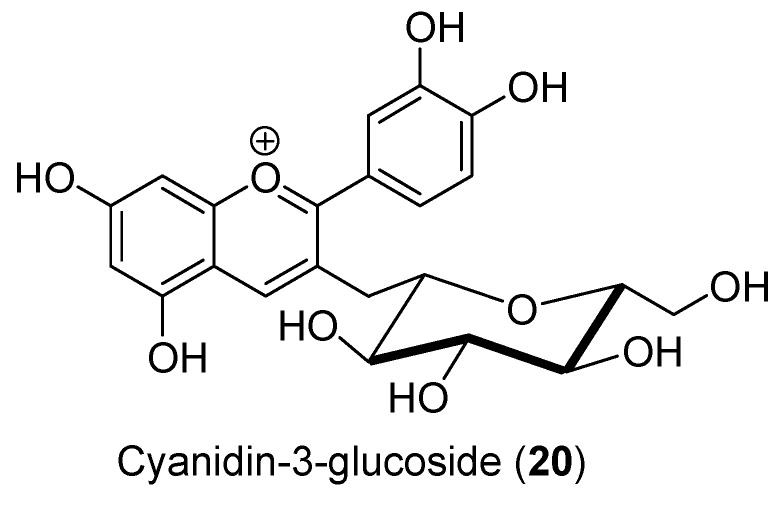
Structure of cyanidin-3-glucoside (**20**).

Anthocyanidins are colored pigments highly abundant in berries, currants, grapes, and tropical fruits. They have antioxidant and antimicrobial properties [87]. Although these flavonoids have not been specifically studied for their potential to regulate testosterone production, they may improve steroidogenesis as they are known to inhibit COX2 activity and to modulate the activity of the MAPK pathway [88,89], both of which are known to influence *StAR* expression and activity in Leydig cells. Interestingly, lead (Pb) mediated decrease in progesterone production from R2C Leydig cells was prevented by cyanidin-3-glucoside (**20**, Figure 9) by protecting mitochondrial function and increasing steroidogenic gene (*StAR*, *Hsd3b,* and *Cyp11a1*) expression [90]. In addition, this anthocyanin also upregulated the activities of the MAPK and PKA signalling pathways [90], promoting steroid production. Cyanidin-3-glucoside (**20**) was also reported to improve the expression levels of steroidogenic proteins (StAR, Hsd3b, and Cyp11a1) and of the LH receptor within the testes of mice exposed to cadmium, an important neuroendocrine disruptor [91]. Thus, anthocyanidins may promote testosterone production by Leydig cells through their antioxidant properties.

Considering the entire data reported on the regulation of steroidogenesis by flavonoids and isoflavonoids, a structure-activity relationship may explain the differences in inhibition and activation of androgen production. Indeed, isoflavonoids with the phenol group in the position 3 of cycle C, such as isoflavones (daidzein (**5**) and genistein (**6**)), have been suggested to preferentially inhibit HSD3B2 and HSD17B3 rather than aromatase enzyme activities [92]. This may explain the more potent inhibitory action of isoflavones on testosterone production from H295R adrenal cells, compared to a flavonoid such as apigenin (**2**) which has its phenol group in the position 2 of cycle C [30].

Aging is associated with reduced StAR protein levels within adult Leydig cells, resulting in defective mitochondrial cholesterol import and lower testosterone production [93,94]. As previously reported [95], age-related decline in testosterone production may be delayed by increasing *StAR* and/or *Cyp11a1* gene expressions using supplementation with flavonoids or chemical derivatives. In aging males, Cox2 activity is increased in Leydig cells, resulting in decreased *StAR* gene expression and testosterone production [28]. Interestingly, flavonoids such as chrysin (**1**), apigenin (**2**), luteolin (**3**), and quercetin (**7**) can promote *StAR* expression and steroidogenesis by inhibiting Cox2-dependent signalling. Therefore, the intake of these flavonoids may delay age-related decline in testosterone production in males.

## 4. Conclusions

**Figure 10:**
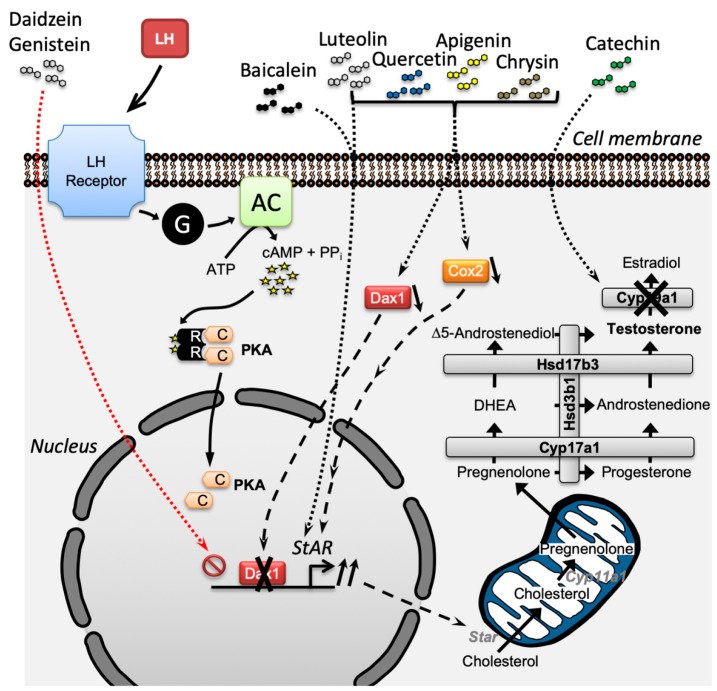
Summary of the mechanism of action of flavonoids and isoflavonoids on testosterone production from testicular Leydig cells. Flavonoids and isoflavonoids mainly regulate steroidogenesis through the modulation of *StAR* gene expression. Dotted lines represent the unknown regulatory mechanisms of flavonoids and isoflavonoids.

Importantly, plasma concentrations of flavonoids for an adequate response of testicular Leydig cells are in the low micromolar range which can be attained with high quality nutrition mainly composed of fruits and vegetables. In mammals, appropriate Sertoli cell function is highly dependent on testicular androgen levels to support normal spermatogenesis. According to the cumulated literature and our data, flavonoids having a 5,7-dihydroxychromen-4-one backbone tend to increase StAR expression, contributing to increased testosterone production from testis Leydig cells (Figure 10). Although numerous polyphenols demonstrate either activation or inhibition effects on androgen biosynthesis, consideration should be taken for combined exposure to naturally occurring flavonoids and isoflavonoids and their potential additive or synergistic effects on steroidogenesis.
